# The Isopropyl Gallate Counteracts Cyclophosphamide-Induced Hemorrhagic Cystitis in Mice

**DOI:** 10.3390/biology11050728

**Published:** 2022-05-09

**Authors:** Lucas Solyano Almeida de Oliveira, Sara Raquel de Moura Bandeira, Rodrigo Lopes Gomes Gonçalves, Benedito Pereira de Sousa Neto, Diana Carvalho de Rezende, Antonio Carlos dos Reis-Filho, Ian Jhemes Oliveira Sousa, Flaviano Ribeiro Pinheiro-Neto, Boris Timah Acha, Gabriela do Nascimento Caldas Trindade, Lázaro Gomes do Nascimento, Damião Pergentino de Sousa, Fernanda Regina de Castro Almeida, Massimo Lucarini, Alessandra Durazzo, Daniel Dias Rufino Arcanjo, Francisco de Assis Oliveira

**Affiliations:** 1 Medicinal Plants Research Center, Federal University of Piauí, Av. Nossa Senhora de Fátima s/n, Teresina 64049-550, Brazil; lucas.solyano@gmail.com (L.S.A.d.O.); sararaquel.sr@hotmail.com (S.R.d.M.B.); profrodrigolgoncalves@gmail.com (R.L.G.G.); bneto100@hotmail.com (B.P.d.S.N.); diana-rezende@hotmail.com (D.C.d.R.); carlosfilho_089@hotmail.com (A.C.d.R.-F.); ianjhemes@gmail.com (I.J.O.S.); flavianopinheiro993@gmail.com (F.R.P.-N.); timah.boris@yahoo.com (B.T.A.); gabrielacaldas.gc@gmail.com (G.d.N.C.T.); ferecal@ufpi.edu.br (F.R.d.C.A.); fassisol@ufpi.edu.br (F.d.A.O.); 2 Laboratory of Pharmaceutical Chemistry, Federal University of Paraíba, João Pessoa 58051-900, Brazil; lazarofarm2@gmail.com (L.G.d.N.); damiao_desousa@yahoo.com.br (D.P.d.S.); 3 CREA-Research Centre for Food and Nutrition, Via Ardeatina 546, 00178 Rome, Italy; massimo.lucarini@crea.gov.it (M.L.); alessandra.durazzo@crea.gov.it (A.D.); 4 Laboratory of Functional and Molecular Studies in Physiopharmacology, Department of Biophysics and Physiology, Federal University of Piauí, Teresina 64049-550, Brazil

**Keywords:** hemorrhagic cystitis, ifosfamide, isopropyl gallate, antineoplastic chemotherapy

## Abstract

**Simple Summary:**

Ifosfamide (IFOS) is an antineoplastic drug used to treat various cancers. Hemorrhagic cystitis (HC) is the main adverse effect related to the use of IFOS. Acrolein, a metabolite of IFOS, is the main molecule responsible for this side-effect, resulting in increased oxidative stress and production of inflammatory mediators, which culminate in the degradation of bladder tissue. In clinical practice, mesna is used to reduce the incidence of HC. However, this drug presents some dose-dependent side effects. Isopropyl gallate (IPG), a gallic acid-derived compound, is promising for the development of new anti-inflammatory agents. In the present study, the effects of oral administration of IPG against IFOS-induced hemorrhagic cystitis in mice were investigated. Interestingly pretreatment of IPG was able to reduce IFOS-induced bladder damage evidenced by a decrease of inflammatory parameters and improvement in histology and antioxidants of the urinary bladder. Furthermore, IPG significantly decreased the expression levels of inflammatory biomarkers in the bladder tissue. These preliminary findings suggest that IPG represents a promising adjuvant therapy for protecting against IFOS-induced urotoxicity.

**Abstract:**

Hemorrhagic cystitis is the main adverse effect associated with the clinical use of oxazaphosphorine, resulting in increased oxidative stress and proinflammatory cytokines, which culminate in injury of the bladder tissue. The aim of this study was to evaluate the protective effect of isopropyl gallate (IPG) against ifosfamide (IFOS)-induced hemorrhagic cystitis in mice. The induction of the hemorrhagic cystitis model was carried out using a single dose of IFOS (400 mg/kg, i.p.) four hours after oral pretreatment with IPG (6.25, 12.5, 25, and 50 mg/kg) or saline (vehicle). Mesna (positive control; 80 mg/kg, i.p.) was administered four hours before and eight hours after induction of cystitis. In the present study, IPG 25 mg/kg significantly decreased edema and hemorrhage, with a reduction of the bladder wet weight (36.86%), hemoglobin content (54.55%), and peritoneal vascular permeability (42.94%) in urinary bladders of mice. Interestingly, IPG increased SOD activity (89.27%) and reduced MDA levels (35.53%), as well as displayed anti-inflammatory activity by decreasing TNF-α (88.77%), IL-1β (62.87%), and C-reactive protein (56.41%) levels. Our findings demonstrate that IPG has a substantial protective role against IFOS-induced hemorrhagic cystitis in mice by enhancing antioxidant activity and proinflammatory mechanisms. Thus, IPG represents a promising co-adjuvant agent in oxazaphosphorine-based chemotherapy treatments.

## 1. Introduction

Oxazaphosphorines are DNA-alkylating chemotherapeutic agents (nitrogen mustard) which have cytotoxic and antiproliferative action. Cyclophosphamide (CYP) and ifosfamide (IFOS) (oxazaphosphorins) have been the most used cytotoxic drugs since they are effective against several types of both benign and malignant neoplastic diseases [1]. Cancers treated by these drugs include those affecting children and adults [2]. Ifosfamide is widely utilized against testis cancer, lung cancer, soft tissue tumors, ovary carcinoma, osteosarcomas, and lymphomas, and its effects are usually more aggressive compared with cyclophosphamide, with fewer myelotoxic effects [3].

Hemorrhagic cystitis (HC) represents one of the most aggressive conditions noticed in patients after conventional chemotherapy regimens with IFOS and cyclophosphamide (CYP). HC is a consequence of the activation of inflammatory molecular mechanisms that lead to a diffuse reaction of cell death: pyroptosis. Pathogens (bacteria or viruses), ionizing radiation, or acrolein, a urinary metabolite of cyclophosphamide or ifosfamide, have been reported as the major causes of pyroptosis in the bladder [4,5].

The principal mechanism involving IFOS-induced bladder inflammation has to do with the direct contact between uroepithelium and acrolein, where acrolein is a urotoxic metabolite of ifosfamide. Several proinflammatory factors, such as cytokines (e.g., TNF-α and IL-1β), have been widely associated with pathogenesis of a damaged bladder epithelium. These factors are mainly associated with the deleterious action of metabolites such as acrolein, a byproduct of degradation of the oxazaphosphorine [6], which is filtered by the kidneys and concentrated in the bladder. This active metabolite can lead to intense apoptosis/necrosis in the urothelium, which in turn leads to bladder ulceration and edema [2]. Acrolein is also responsible for the activation of intracellular reactive oxygen species and the formation of nitric oxide, leading to the increase of peroxynitrite, a potent oxidant which leads to lipid peroxidation [7].

Attempts have been made to alleviate the adverse effects of IFOS on the bladder mucosa. For example, before initiation of treatment with cyclophosphamide, Mesna (2-mercaptoethane sulfonate sodium) prevented hemorrhagic cystitis via neutralization of acrolein. However, Mesna has no effect in the treatment of hemorrhagic cystitis after its onset, and it has been reported to be ineffective in relieving some clinical symptoms. Moreover, Mesna can promote some adverse effects, such as allergic reactions, dermatosis, and hypersensitivity in the skin [8]. Continuous bladder irrigation leading to blood clot evacuation, instillation of alum or formalin, fulguration using electrocautery, hyperbaric oxygen therapy, and in extreme conditions, cystectomy with urinary diversion, represent alternative treatment modalities of hemorrhagic cystitis [9]. In the literature, it is possible to find a tendency towards natural products as alternative safe therapies to reduce the urotoxicity caused using oxazaphosphorines [10,11,12,13].

Gallic acid is derived from the shikimic acid pathway, an intermediate of secondary metabolism, and is a component of plant-derived hydrolysable tannins [14]. According to Correia et al. [15], several evidences are reported on the use of gallic acid in the treatment of microalbuminuria, and selective cytotoxicity against a variety of cell tumors. It is noteworthy that it has already been described that gallic acid derivatives can exert an anti-inflammatory effect by decreasing neutrophil migration and stabilizing mast cell degranulation [16] and antioxidant, antimicrobial, and antimutagenic properties [14,16].

The low oral bioavailability of gallic acid has prompted research on the development of synthetic-derived compounds. Among them, isopropyl gallate (IPG) is potentially useful in the development of new anti-inflammatory agents, as reported by the recent review carried out by Bai et al. [17], which summarizes the IPG-induced pharmacological activities and molecular mechanisms involved in inflammation-related diseases. Furthermore, there was no experimental or clinical study in which isopropyl gallate was used to treat IFOS-related HC in the literature review. In this context, the present work is conducted to evaluate the anti-inflammatory activity of isopropyl gallate in an experimental model of IFOS-induced HC in mice.

## 2. Materials and Methods

### 2.1. Synthesis of Isopropyl Gallate (IPG)

Concentrated H_2_SO_4_ (0.5 mL) was added to a mix of gallic acid (0.1 g, 0.59 mmol) and isopropyl alcohol (10 mL). The resulting mixture was stirred by refluxing for 24 h and monitored by thin-layer chromatography. Upon completion, excess alcohol was evaporated by reduced pressure and the crude product was diluted into ethyl acetate (10 mL) and then washed with 15 mL of water. After the separation of the organic phase, the extraction of aqueous phase was carried out utilizing ethyl acetate (3 × 10 mL) and then the combined organic phase was dried with anhydrous sodium sulfate (Na_2_SO_4_), then filtered, and evaporated be means of reduced pressure. Pure isopropyl gallate (0.0976 g, 0.45 mmol) was obtained in 78% yield by using silica gel column chromatography with the elution with hexane:ethyl acetate (1:1) [18].

### 2.2. Experimental Animals and Euthanasia

Male albino (Swiss) mice, weighing 25 to 30 g, were distributed into 7 groups containing each one 6 animals. The animals were obtained from the Central Bioterium of the Federal University of Piauí. The animals were kept at a temperature of 24 ± 1 °C and maintained on a 12 h light–dark cycle with water and food ad libitum, and they were fasted for 6 h before the experiments.

Euthanasia procedures were performed intraperitoneally using an anesthetic overdose (sodium thiopental 150 mg/kg and lidocaine 10 mg/kg), under the supervision of the veterinarian Adeline de Andrade Carvalho (CRMV-PI 866-VP) in accordance with CONCEA guidelines in paragraph 1, Article 14 of CFMV Resolution No. 1000 of 11 May 2012. This study was submitted and approved by the Ethics Committee for Animal Experimentation—CEUA/UFPI, protocol No. 453/17.

### 2.3. Experimental Model of Induction of Hemorrhagic Cystitis with Ifosfamide

**Figure 1:**
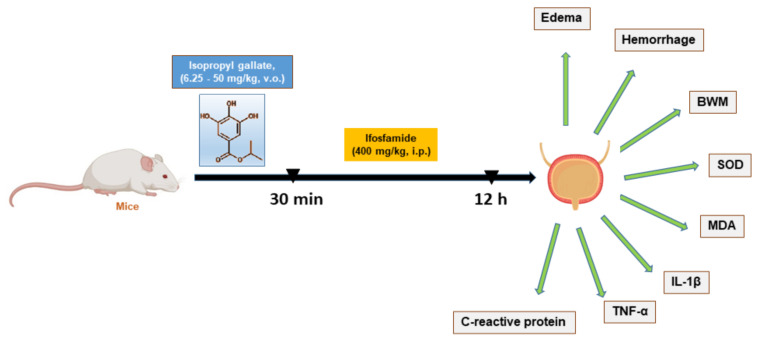
Diagrammatic representation of the investigation of IPG in ifosfamide-induced hemorrhagic cystitis.

Animal groups were pretreated as follows, except for group I (Sham) which did not receive any treatment: Group II, the negative control (NC), were pretreated with vehicle (Tween-saline), and group III, the positive control, were treated with sodium 2-mercaptoethane sulfonate (Mesna) at 80 mg/kg intraperitoneally, three times at intervals of 4 h because its plasma half-life is short (approximately 1.5 h). Meanwhile, groups IV, V, VI, and VII were pretreated orally with isopropyl gallate (IPG) at single doses of 6.25, 12.5, 25, and 50 mg/kg, respectively, which was prepared by dissolving IPG 0.25% *w*/*v* in DMSO 0.1% and diluting with NaCl 0.9%. After 30 min, a single dose of ifosfamide (400 mg/kg, i.p.) was administered to all animal groups except group I (Sham) to induce hemorrhagic cystitis, and 12 h later, animals were euthanized by an anesthetic overdose (sodium thiopental 150 mg/kg and lidocaine 10 mg/kg, intraperitoneally). The experimental design of this study consisted of pretreatment with isopropyl gallate (IPG), and later, induction of hemorrhagic cystitis [19], as illustrated in Figure 1.

### 2.4. Evaluation of the Bladder Tissue Wet Weight

After euthanasia, the bladders were removed, emptied, and weighed using an analytical balance. An increase of bladder wet weight (BWW) was considered as indicative of bladder edema, and was expressed as bladder mass/20 g of animal weight after treatment.

### 2.5. Evaluation of Macroscopic Parameters

A gross pathologic evaluation was performed following the guidelines established by Gray et al. [20], whereby semi-quantitative assessment parameters are defined based on observations of macroscopic morphological states.

### 2.6. Histopathological Analysis

After euthanasia, bladders were removed, emptied, and fixed in 10% buffered formalin (pH~7.2). After 48 h, the samples were processed by dehydration in increasing gradients of alcohol and xylol. Afterwards, the samples were embedded in liquid paraffin, and sectioned using a 4 μm-thick microtome. Then, sections were rehydrated and stained with Hematoxylin and Eosin (H&E). The preparations were analyzed according to the parameters defined by Gray et al. [20], using a semi-quantitative scale to analyze the data presented below.

### 2.7. Quantification of Bladder Hemoglobin by the Cyanmethemoglobin Method

To quantify blood loss in the bladder tissue, a colorimetric test was used. This method is based on the iron (Fe^2+^) oxidation of the hemoglobin molecule by potassium ferricyanide in a weak alkaline solution, forming methemoglobin, which is converted to cyanmethemoglobin after reaction with potassium cyanide, producing a reddish color proportional to the hemoglobin concentration in the sample. In this test, the kit (Labtest, Minas Gerais, Brazil) consisting of a formulation of the reagent made by Harold and Drabkin [21] was adapted and used.

### 2.8. Evaluation of Vascular Protein Leakage by Evans Blue Dye Technique

In this assay, Evans blue (25 mg/kg) was intravenously administered via caudal plexus 30 min before euthanasia. Thereafter, bladders were removed, dissected, incubated with a formamide solution (1 mL/bladder) at 56 °C for 6 h (overnight), and then submitted to dye extraction. The total amount of extracted dye was evaluated by measuring absorbances at 550 nm using a microplate reader (EZ Read 400, Biochrom, Cambridge, England). At the same time, an absorbance–concentration curve was plotted. Bladder wet weight was measured and expressed in ng of Evans blue/mg of tissue (mean ± SEM) [22].

### 2.9. Determination of Superoxide Dismutase (SOD) Activity in Bladder Tissue

The activity of superoxide dismutase was determined following a modified spectrophotometric assay [23]. In this methodology, enzymatic activity is calculated from the amount of SOD that has the ability of inhibiting the formation of nitrite by 50%. In this order, the bladder tissue was homogenized in a potassium phosphate buffer (50 nM, pH 7.4) at a ratio of 100 mg/mL (*w*/*v*). Then, 100 microliters of homogenate were added to 1110 μL of phosphate buffer, 75 μL of L-methionine (20 mM), 40 μL of Triton X-100 (1% *v*/*v*), 75 μL of hydroxylamine chloride (10 mM), and 100 μL of EDTA (50 μM). The resulting solution was incubated in a 37 °C water bath for 5 min, and then 80 μL of riboflavin solution (50 μM) was added to it and then exposed to light for 10 min. Next, 100 μL of the sample was removed and another 100 μL of Griess reagent was added to the wells, and after 10 min, the absorbance was read at 550 nm using a microplate reader (EZ Read 400, Biochrom, Cambridge, England).

### 2.10. Determination of Malondialdehyde (MDA) Levels in Bladder Tissue

Bladder tissue MDA levels were evaluated according to Mihara and Uchiyama [24]. The bladders were homogenized in 1.15% ice-cold KCl to prepare a 10% homogenate. Then, 250 µL aliquots of the homogenate were added to tubes with 1.5 mL of 1% H_3_PO_4_ and 500 µL of an aqueous thiobarbituric acid solution (0.6%). Subsequently, the tubes were heated for 45 min in a 100 °C water bath, followed by cooling in an ice bath and the addition of 2 mL of n-butanol. Then, samples were vortexed for 1 min, and then centrifuged at 1500 rpm for 10 min. The absorbance of the supernatant was read at 520 and 535 nm wavelengths using a microplate reader (EZ Read 400, Biochrom, Cambridge, England). Results were expressed in nmol/g of bladder tissue.

### 2.11. Ultra-Sensitive Quantitative Determination of C-Reactive Protein (CRP) in Serum Samples by Immunoturbidimetry

For C-reactive protein determination, blood samples were withdrawn from the retro orbital plexus 30 min before euthanasia, and then serum was obtained by centrifugation. The concentration of C-reactive protein (CRP) was determined using the PCR Plus Ultra Turbiquest (Labtest, Lagoa Santa, Brazil), an immunoturbidimetric method easily applicable to automated analyzers. The absorbances of samples were measured at the wavelength of 540 nm (530 to 550 nm).

### 2.12. Tissue Levels of TNF-α and IL-1β by ELISA

TNF-α and IL-1β were measured in the bladder homogenate samples using specific ELISA kits. The enzyme-linked immunosorbent assay was performed at room temperature (25 ± 5 °C) using 100 µL of each reaction component. TNF-α and IL-1β were determined using the standard kit (R&D Systems, Inc., Minneapolis, MN, USA) according to the manufacturer’s guidelines. Antibodies and other substrates were diluted in BSA (1% bovine serum isolated albumin in PBS, pH 7.4), and 10% of homogenate of bladder tissue was dispersed in 0.5% BSA in PBS (pH 7.4). The absorbances were read at a wavelength of 450 nm in a microplate reader (EZ Read 400, Biochrom, Cambridge, England). Results were expressed as cytokine picograms per milligram of tissue (pg/mg of protein).

### 2.13. Statistical Analysis

The results were expressed as mean ± SEM. The inhibition percentages were calculated as the mean of inhibitions obtained for each individual experiment. Analysis of variance (ANOVA) followed by Bonferroni’s post hoc test was performed to compare the groups. Differences between groups were considered statistically significant when *p* < 0.05, using the GraphPad prism 6 software (GraphPad Software, La Jolla, CA, USA).

## 3. Results

Besides the preliminary evaluation of the physical and chemical parameters of IPG, the anti-inflammatory effect of IPG was examined considering the following parameters: (i) wet weight of the bladder tissue, (ii) macroscopic parameters, (iii) histopathological alterations, (iv) bleeding induced by ifosfamide, (v) vascular protein leakage, (vi) superoxide dismutase (SOD) levels, (vii) malondialdehyde (MDA) levels, (viii) C-reactive protein (CRP) levels, and (ix) TNF-α and IL-1β levels.

### 3.1. Physical and Chemical Parameters of Isopropyl Gallate (IPG)

Isopropyl gallate was prepared from gallic acid via Fischer esterification using isopropyl alcohol and refluxing sulfuric acid [18].

Physical and chemical parameters of gallic acid showed: White solid; MP = 145–1416 °C; TLC (1:1 hexane/EtOAc), R_f_ = 0.87; ^1^H-NMR (400 MHz, DMSO-d_6_), δ_H_ 9.25 (s, 2H, m-OH), 8.89 (s, 1H, p-OH), 6.94 (s, 2H, H-2, H-6), 5.02 (sept, 1H, H-1′), 1.25 (d, J = 4 Hz, 6H, H-2′, H-3′); ^13^C-NMR (100 MHz, DMSO-d_6_), δ_C_ 165.4, 145.6, 138.4, 120.1, 108.6, 67.3, 21.9. IR _Vmax_ (KBr, cm^−1^) 3336, 3109, 2963, 1690, 1616, 1467, 1310.

### 3.2. Effects of IPG on Wet Weight of the Bladder Tissue

**Figure 2:**
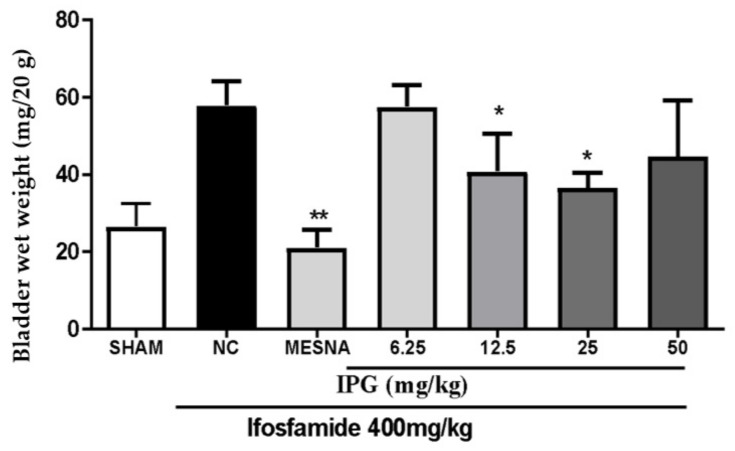
Effects of IPG on bladder wet weight in ifosfamide-induced hemorrhagic cystitis. Data are expressed as mean ± SEM (*n* = 6) of the ratio of bladder wet weight to animal mass after the experimental protocol (mg/20 g of animal weight). The values were compared with the negative control (NC, Tween/Saline), by one-way analysis of variance (ANOVA) followed by the Bonferroni test; * *p* < 0.05, ** *p* < 0.001.

Figure 2 shows that the administration of ifosfamide at a dose of 400 mg/kg led to a marked increase of the wet weight of the bladders (BWW). Regarding the effects of IPG, the results showed that the administration of IPG at the doses of 12.5 and 25 mg/kg significantly decreased the wet bladder weight, by 29.73% and 36.86%, respectively, when compared with the negative control. Besides, a loss of activity with lower and higher doses was observed, whereby 6.25 and 50 mg/kg of IPG did not show any activity on this parameter. Pretreatment with Mesna significantly inhibited the increase in BWW, guaranteeing a remarkable protective effect.

### 3.3. Effects of IPG on Macroscopic Parameters

**Figure 3:**
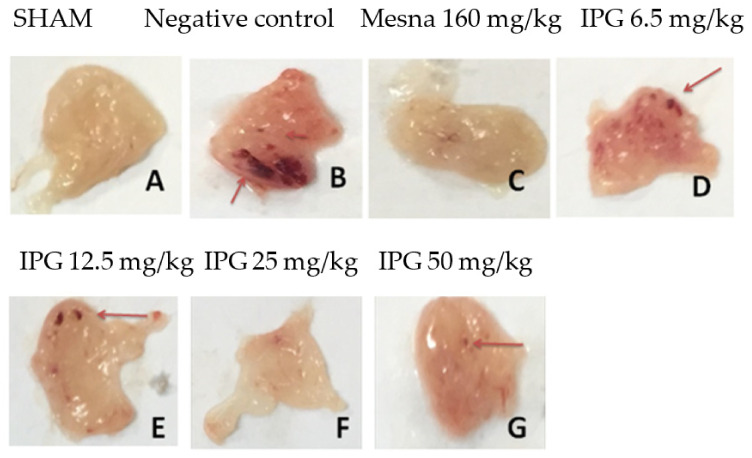
Effects of IPG on macroscopic parameters of hemorrhagic cystitis induced by ifosfamide. (**A**) Group “Sham” received only saline as a placebo, without induction of hemorrhagic cystitis. (**B**) The negative control group were treated with the vehicle (Tween/saline) before induction of hemorrhagic cystitis. (**C**) The positive control group were treated intraperitoneally with Mesna (2-mercaptoethanesulfonate sodium) before induction of hemorrhagic cystitis. Meanwhile, (**D**–**G**) the other groups were orally pretreated with IPG (6.25, 12.5, 25, and 50 mg/kg, respectively) before induction of hemorrhagic cystitis. The choice of the above images was based on the selection of a bladder considered the median among each group.

Figure 3 shows macroscopic images of the mice bladders after ifosfamide-induced hemorrhagic cystitis. It was observed that the bladders in physiological conditions (Figure 3A) have little vascularization, besides a smooth appearance and characteristic color. However, in the bladder tissue with hemorrhagic cystitis, the macroscopic changes were quite severe (Figure 3B), and the bladder presented with visible edema and multiple points of apparent hemorrhage. The use of Mesna was able to markedly reduce the injury (Figure 3C). Similarly, pretreatment with IPG attenuated the apparent lesions, especially at doses of 12.5 and 25 mg/kg (Figure 3E,F). On the other hand, IPG at doses of 6.25 or 50 mg/kg failed to inhibit the damage caused by IFOS.

### 3.4. Effects of IPG on Histopathological Changes

**Figure 4:**
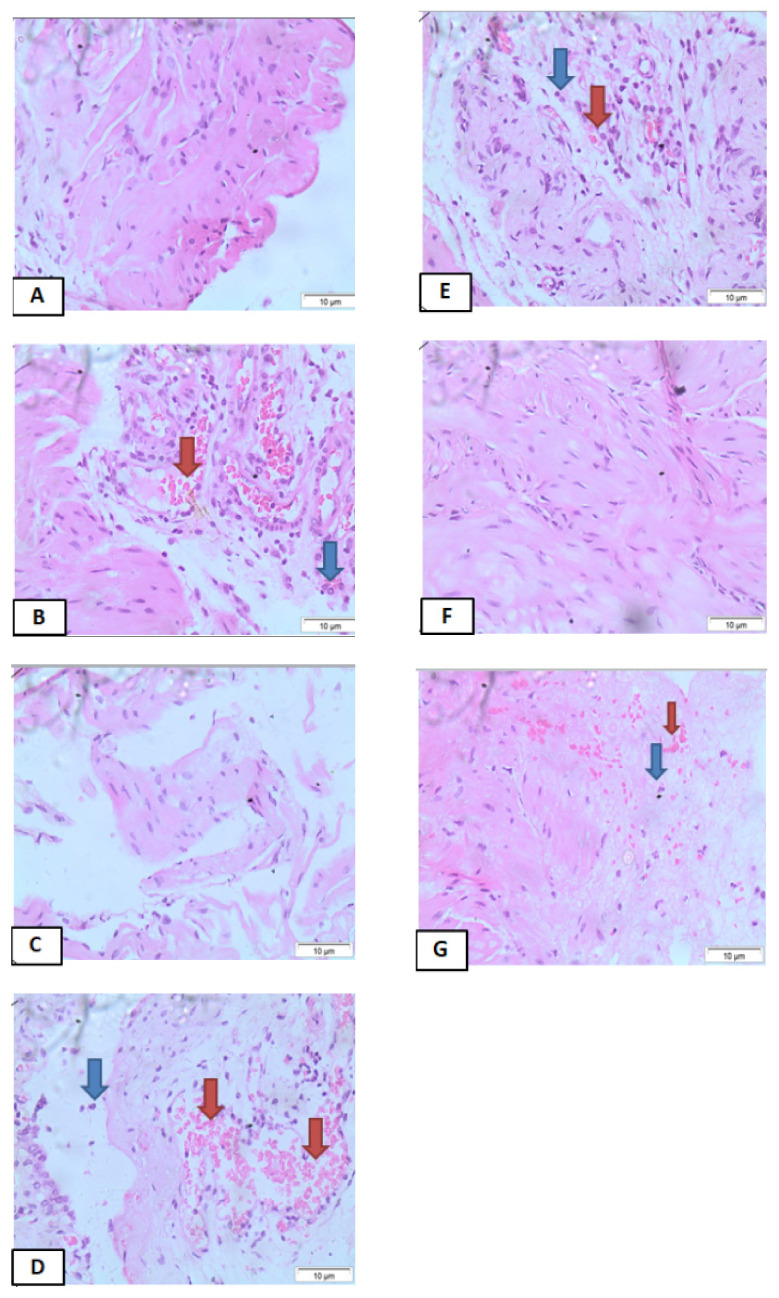
Effects of IPG on histopathological changes in bladder tissues of mice with ifosfamide-induced hemorrhagic cystitis. Representative histological alterations of the bladders of the mice of different groups. (**A**) Control group (Sham). (**B**) IFOS group. (**C**) IFOS + Mesna (160 mg/kg) group. (**D**–**G**) Test groups IFOS + IPG (6.25, 12.5, 25, or 50 mg/kg, respectively). The arrows indicate: hemorrhage (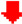) and nucleated polymorphs (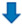) (Hematoxylin and Eosin staining, ×400 magnification).

Based on the histological analysis, the administration of IFOS induced severe bladder changes (e.g., edema, bleeding points, cell loss, and infiltration of polymorphous nuclear cells) (Figure 4B). The Mesna group (Figure 4C) demonstrated a marked reduction in cell losses, hemorrhage, and edema compared to the control group (Sham) (Figure 4A). The groups IPG 6.25 mg/kg (Figure 4D), 12.5 mg/kg (Figure 4E), and 50 mg/kg (Figure 4G) showed a slight reduction in cell losses, hemorrhage, and edema, compared to the negative control group. Treatment with IPG 25 mg/kg (Figure 4F) or Mesna (80 mg/kg) (Figure 4C) led to a partial recovery of urothelium, lesser inflammatory cell infiltrate, fewer mucosal ulcerations and fibrosis, and mild to moderate edema and hemorrhage.

### 3.5. Effect of IPG on Bleeding Induced by Ifosfamide

**Figure 5:**
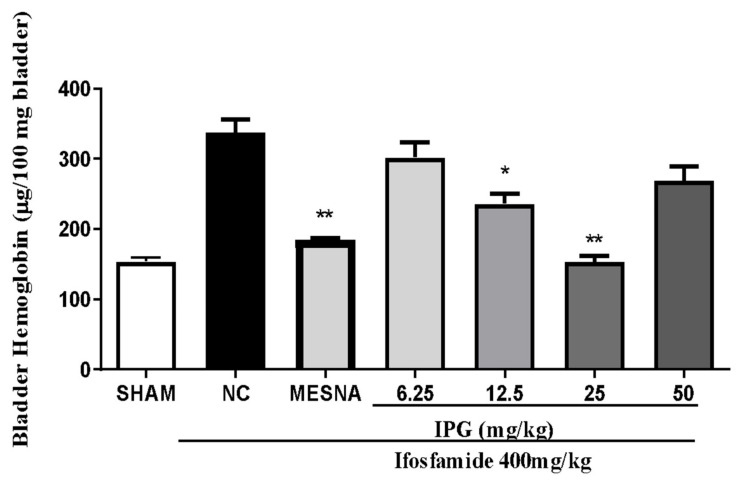
Effect of IPG on decreased bladder hemoglobin levels in animals with ifosfamide-induced hemorrhagic cystitis. Data are expressed in µg of hemoglobin per 100 mg of tissue in the mean ± SEM format (*n* = 6). Data were compared with the negative control (Tween/saline), by one-way analysis of variance (ANOVA), followed by the Bonferroni test; * *p* < 0.05, ** *p* < 0.001.

Unlike the Sham group, bladders from IFOS-treated animals demonstrated a significant increase of hemorrhage, as reported in Figure 5. Pretreatment with IPG (12.5 or 25 mg/kg) showed significant differences when compared with the negative control (IFOS group), with a reduction of 30.1% and 54.55%, respectively. On the other hand, IPG at doses of 6.25 or 50 mg/kg was unable to significantly reduce hemorrhage. Intraperitoneal treatment with Mesna (160 mg/kg) significantly inhibited hemorrhage. No significant changes were revealed in mice treated with Mesna or IPG 25 mg/kg.

### 3.6. Effects of IPG on Vascular Protein Leakage

**Figure 6:**
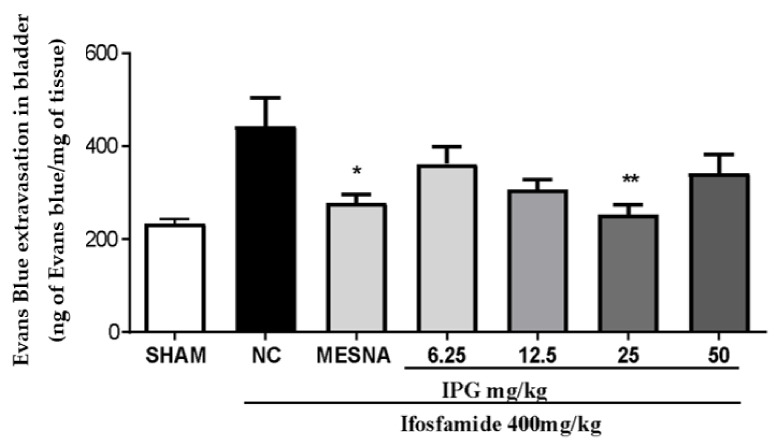
Effect of IPG on vascular leakage of Evans blue (EB) in bladders of animals with ifosfamide-induced hemorrhagic cystitis. Data are expressed in ng of Evans blue per mg of bladder as mean ± SEM (*n* = 6). Data were compared with the negative control (Tween/saline), by one-way analysis of variance (ANOVA), followed by the Bonferroni test; * *p* < 0.05, ** *p* < 0.001.

As reported in Figure 6, IFOS induced a significant increase in plasma leakage in the mice urinary bladder. IPG, at a dose of 25 mg/kg, was able to reduce the vascular protein leakage in the bladder tissue of Evans blue by 42.94%. Mesna reduced vascular protein leakage by 36.28% when compared to the NC (IFOS group), considering *p* < 0.05.

### 3.7. Effect of IPG on Superoxide Dismutase (SOD) Activity

**Figure 7:**
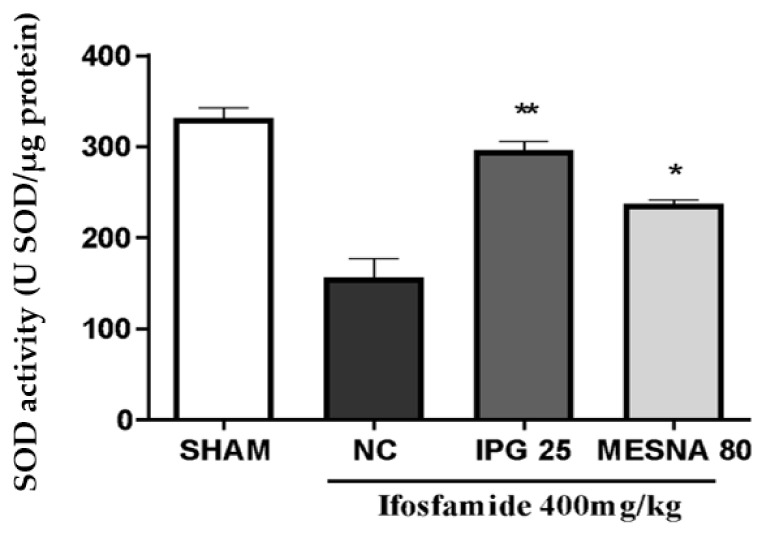
Effect of IPG on superoxide dismutase (SOD) levels in the bladder tissue of animals with ifosfamide-induced hemorrhagic cystitis. Data are expressed in U of SOD per µg of protein, as mean ± SEM (*n* = 6). Data were compared with the negative control (NC, Tween/saline) by one-way analysis of variance (ANOVA), followed by the Bonferroni test; * *p* < 0.05, ** *p* < 0.001.

A marked reduction in bladder SOD activity by 47.26% was reported for the negative control group when compared to the Sham group. Interestingly, IPG at a dose of 25 mg/kg markedly increased the SOD activity in the bladder tissues (89.27%). This effect was significantly higher than that observed for the Mesna group (80 mg/kg), when compared with the negative control (71.62%) (Figure 7).

### 3.8. Effect of IPG on Malondialdehyde (MDA) Levels

**Figure 8:**
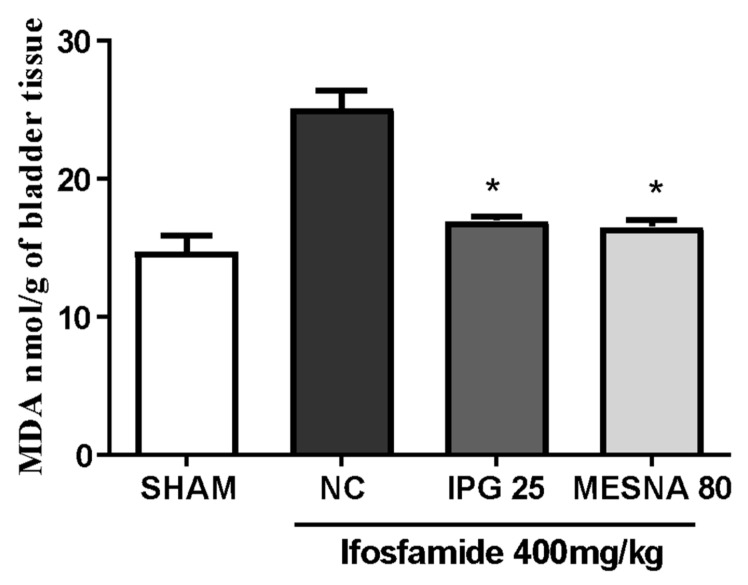
Effect of IPG on bladder MDA levels in animals with ifosfamide-induced hemorrhagic cystitis. Data are expressed in nmol of MDA per g of tissue, as mean ± SEM (*n* = 6). Data were compared with the negative control (Tween/saline) by one-way analysis of variance (ANOVA), followed by the Bonferroni test; * *p* < 0.05.

Figure 8 shows that the administration of ifosfamide led to a significant increase in the levels of MDA in the urinary bladder when compared with the Sham group. The pretreatment with IPG (25 mg/kg) or Mesna (80 mg/kg) significantly reduced MDA levels (32.53% and 34.29%, respectively) when compared with the negative control group (*p* < 0.05).

### 3.9. Effect of IPG on C-Reactive Protein (CRP)

**Figure 9:**
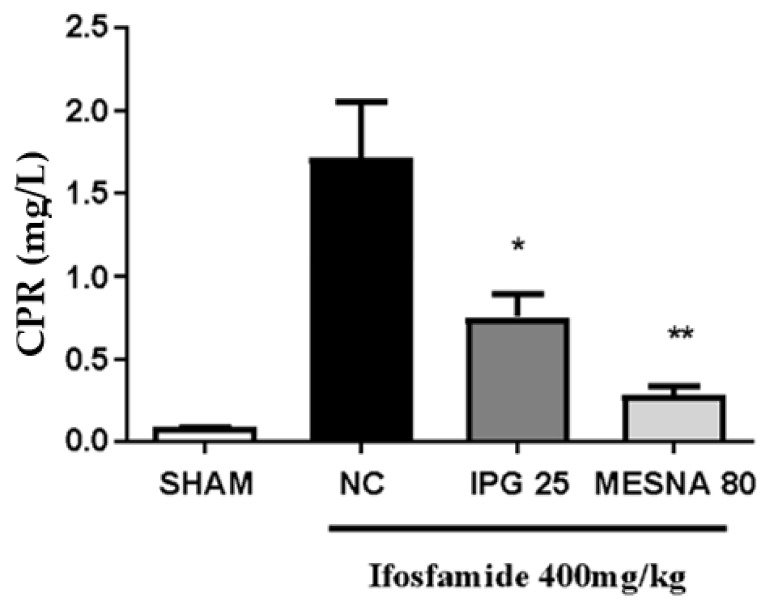
Effect of IPG on ultra-sensitive C-reactive protein (CRP) quantitatively determined in serum samples by immunoturbidimetry. Data are expressed in mg of CRP per liter of serum, as mean ± SEM (*n* = 6). Data were compared with the negative control (NC, Tween/saline) by one-way analysis of variance (ANOVA), followed by the Bonferroni test; * *p* < 0.05, ** *p* < 0.001.

As shown in Figure 9, serum CRP levels increased in the negative control group as an effect of IFOS when compared with the Sham group. Besides, after treatment with IPG at the dose of 25 mg/kg, CRP levels significantly reduced when compared with the negative control group (mean difference of 56.41%), whereas the CRP levels of the Mesna group decreased by 83.68%, considering *p* < 0.05.

### 3.10. Effect of IPG on TNF-α and IL-1β Levels

**Figure 10:**
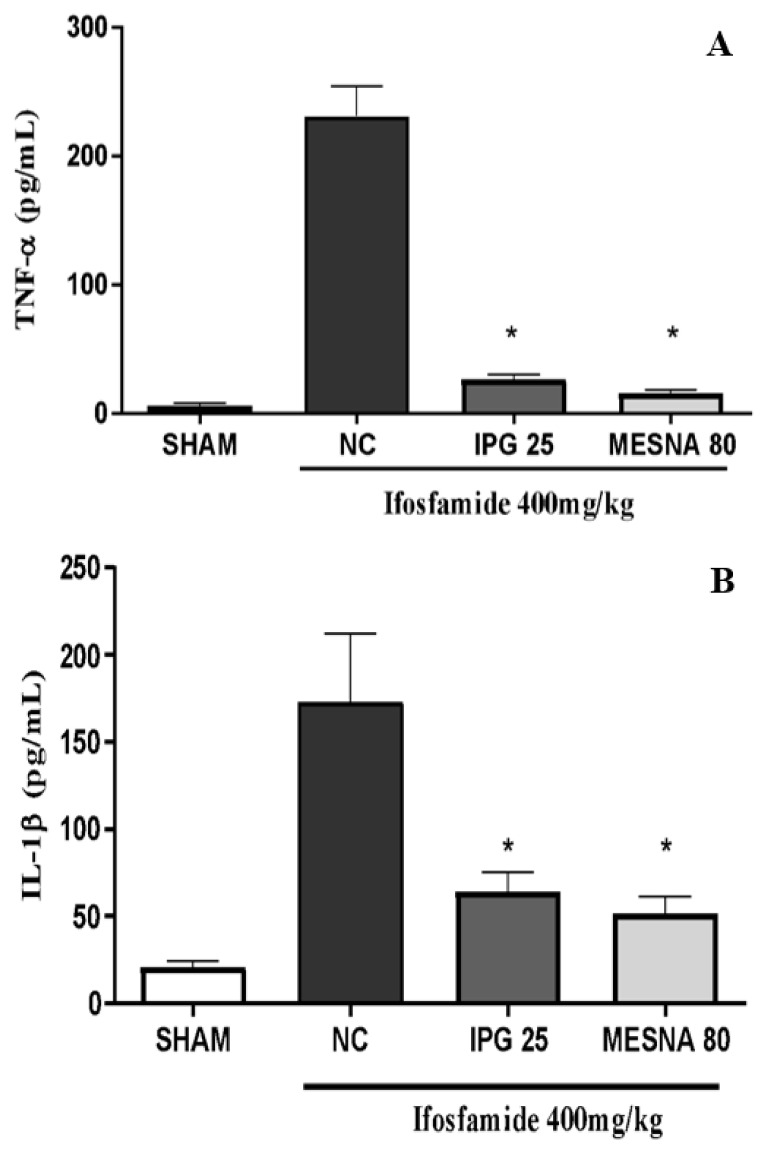
Effect of IPG on TNF-α (**A**) and IL-1β (**B**) levels in the bladder tissue of animals with ifosfamide-induced hemorrhagic cystitis. Data are expressed in pg of TNF-α (**A**) or IL-1β (**B**) per mL of bladder tissue homogenate in the mean ± SEM format (*n* = 6). Data were compared with the negative control (NC, Tween/saline) by one-way analysis of variance (ANOVA), followed by the Bonferroni test; * *p* < 0.05.

The hemorrhagic cystitis induced by IFOS administration also caused a marked increase of TNF-α and IL-1β cytokines when compared with the Sham group. IFOS treatment increased bladder levels of TNF-α when compared with the Sham group. Interestingly, animals pretreated with IPG at a dose of 25 mg/kg showed significantly decreased TNF-α (88.72%) and IL-1β (62.87%) levels when compared with the negative control group (*p* < 0.05). Likewise, the Mesna group showed a reduction of 93.44% and 70.04% in TNF-α and IL-1β levels, with a reduction of 70.04% when compared to the negative control group (Figure 10B) (*p* < 0.05).

## 4. Discussion

Conventional chemotherapy including oxazaphosphorine alkylating agents, e.g., ifosfamide and cyclophosphamide, is frequently related to serious adverse effects, where urological toxicity, especially manifested as hemorrhagic cystitis (HC), represents the most clinically relevant event. The mechanism underlying HC induced by oxazaphosphorine is complex and multimodal. However, the etiology of this devastating complication is associated with the metabolism of oxazaphosphorine, which leads to the biosynthesis of highly reactive metabolites, such as acrolein and chloroacetaldehyde [25].

Acrolein, the main cytotoxic metabolite of oxazaphosphorines, is able to promote the rupture of the intraluminal membrane, making possible its contact with the deeper epithelium layers. Moreover, it causes the displacement of urothelial cells to develop a typical robust inflammatory process, leading to subepithelial edema, neutrophil infiltration, hemorrhage, and endothelial tissue destruction [26,27,28]. This toxic metabolite can directly cleave proteins and break strands of DNA given that it has a reactive unsaturated aldehyde residue, causing cell death. Additionally, acrolein increases reactive oxygen and nitrogen species, leading to damage in protein, DNA, and lipids [7,9]. It is worth mentioning that chloroacetaldehyde, not just acrolein, may also contribute to the urotoxicity (side effects) of ifosfamide [29,30].

The prophylactic administration of Mesna has been shown to be a useful measure for preventing HC, and a great percentage of patients under treatment with oxazaphosphorine agents will develop HC [31]. Furthermore, the treatment with Mesna might be associated with remarkable side effects, such as hypersensitivity-like cutaneous and systemic reactions [32]. Therefore, the search for novel uroprotective agents is required for managing this condition. In this sense, a previous study from our research group reported the anti-inflammatory effects of the monoterpene α-phellandrene, which exerts anti-inflammatory effects and decreases urothelial damage in IFOS-induced hemorrhagic cystitis [13].

The formation of the characteristic edema can be observed in our study, where indirect analysis of the mean bladder wet mass in the negative control group (IFOS) showed a considerable increase when compared to the Sham group. In this experimental model, Mesna and isopropyl gallate (IPG) demonstrated the ability to prevent the onset of edema. Still, in the context of inflammatory induction, in contrast to the results observed in the macroscopic and microscopic parameters, IPG (12.5 and 25 mg/kg) significantly reduced edema, but its activity on hemorrhage was only significant at the dose of 25 mg/kg. These same results can be visually observed through the macroscopic figures that represent the median of each group of the experiment. In this context, a previous work has shown that oral administration of gallic acid prevented the inflammatory signs associated with cyclophosphamide in mice. Furthermore, gallic acid significantly decreased the edema and hemorrhage [33]. Therefore, it is possible that IPG is endowed with anti-inflammatory properties by reducing the parameters of urinary bladder toxicity induced by IFOS. Furthermore, it is worth pointing out that IPG at lower (6.25 mg/kg) or the highest (50 mg/kg) doses does not prevent tissue damage caused by ifosfamide. These profile changes are characteristic of a phenomenon called hormesis, described as an effect of some substances which show biphasic dose responses displaying a “U” shape [34]. This pharmacological response (U-shape) is observed in many herbal medicines, and health benefits of many phytochemicals may also be related to hormetic mechanisms, where a phytochemical can activate different adaptive cellular stress response pathways [35,36].

It is widely known that the oxazaphosphorine-induced hemorrhagic cystitis is the marked presence of a massive extravasation of mature red cells and reticulocytes, characterized by the presence of hematuria [37,38]. This parameter of hemorrhage was approached quantitatively by measuring the tissue hemoglobin, which in turn is directly proportional to the vascular ruptures in the bladder damage caused by the ifosfamide metabolite [39]. The results obtained in this study show that IPG (25 mg/kg) exerted a protective effect against bladder hemorrhage at a dose of 25 mg/kg. Some plant-derived substances have been shown to attenuate this condition, such as ternatin [3], gallic acid [33], S-allylcysteine [39], and oleuropein [25]. This protection may be due to decreased production of proinflammatory cytokines in the bladder which reduce peroxynitrite production [40]. The results of the evaluation of the extent of hemorrhagic cystitis observed by the Evans blue dye dilution method demonstrate that IPG (25 mg/kg) was able to decrease vascular protein leakage and reduce the damage caused by IFOS by 42.94%. These results indicate that the gallic acid derivative may be involved in the protection against the progression of ifosfamide-induced hemorrhagic cystitis. This finding is compatible with the pattern of anti-inflammatory activity demonstrated by phenolic compounds with a gallic acid-related chemical structure found in the literature [41].

Considering the potential antioxidant effects of gallic acid [16,42], we decided to investigate the effect of IPG by determining malondialdehyde (MDA) and superoxide dismutase (SOD) tissue levels in the bladder tissue of animals treated with ifosfamide. Free radicals have the ability to modify reactions that occur in cells and cause deterioration of lipids, proteins, and nucleotides present in tissue cells [43]. The most widely used test for the evaluation of lipid peroxidation is the measurement of tissue MDA concentration, since it is one of the main end products of lipid peroxidation, which is used as a biomarker of cell membrane damage caused mainly by the attack of OH hydroxyl free radicals [43,44]. The investigation of tissue damage resulting from the inflammatory process induced by IFO injury was performed through the measurement of MDA levels. The results showed that pretreatment with IPG significantly decreased levels of MDA in the bladder tissue, compared to the experimental model of hemorrhagic cystitis. These data corroborate findings in the literature, as Wang et al. [45] demonstrated that methyl gallate, as well as some gallic acid metabolites, have high antioxidant activity, evaluated by the presence of thiobarbituric acid reactive substances (TBARS); similarly, it is suggested that this finding is also true for IPG.

It is worth noting that there is a complex endogenous system of defense that protects tissues from cell damage induced by reactive oxygen species and reactive nitrogen species. This antioxidant system can be divided into two: enzymatic, which includes enzymes such as superoxide dismutase (SOD), catalase, glutathione peroxidase, and thioredoxin, and non-enzymatic, including non-enzymatic hydrophilic agents, composed of proteins, uric acid, acid ascorbic, selenium, and zinc, and non-enzymatic lipophilic agents, which include α-tocopherol [46,47]. The antioxidant enzyme SOD deserves more attention as its final enzymatic function results in the reduction of both ROS and RNS. Based on the determination of SOD activity as a biomarker of oxidative stress, it was observed that IPG was able to maintain SOD levels compared to the negative control, showing that isopropyl gallate is able to preserve SOD enzyme activity. In this context, increases in SOD enzyme activity will probably inhibit the formation of reactive oxygen species. These data confirm the findings demonstrated by Wang et al. [46], which showed that gallic acid derivatives recover the SOD enzyme activity in models of oxidative stress in endothelial cells.

Regarding anti-inflammatory activity, it is known that C-reactive protein (CRP) is an acute phase marker of inflammation. It plays a key role in host defense mechanisms against infectious agents and in inflammatory response [48]. Furthermore, CRP promotes the production of proinflammatory cytokines, leading to aggravation of the inflammatory response [49]. IPG (25 mg/kg) decreased CRP levels by 56.41% when compared to the negative control. The reduction in the CRP, MDA, and SOD was possibly due to the regulatory effect of IPG on the inflammatory response.

In the progression of urothelial damage induced by the toxic effect of acrolein, there is an inflammatory stimulus of epithelial cells and conjunctive tissue, as well as tissue macrophages, leading to an increase in inflammatory cytokines at the site of inflammation, mainly tumor necrosis factor alpha (TNF-α) and interleukin 1β (IL-1β) [50]. However, increased TNF-α and IL-1β levels may cause toxic effects, leading to the activation of other cytokines, as well as the oxidative stress-inducing agents, which promote inflammation, activation of neutrophils, amplify and prolong inflammation, as well as stimulating phagocytosis and the production of oxidizing agents, culminating in tissue destruction [38]. Ribeiro et al. [51] demonstrated that these cytokines are involved in urothelial damage and in the inflammatory events leading to hemorrhagic cystitis after ifosfamide administration, apparently by mediating the inflammatory cell migration, edema, and hemorrhage. In this study, IPG proved to reduce TNF-α and IL-1β levels in animals treated with isopropyl gallate, compared with the negative control group. These data were corroborated by macroscopic and histopathological findings related to IPG at the dose of 25 mg/kg. Thus, it is suggested that the beneficial effect of IPG against IFOS-induced urotoxicity may be partly associated with the modulation of the anti-inflammatory response.

In this sense, further investigations are warranted to explore other possible mechanism(s) of the protective action of IPG to evaluate their anti-inflammatory activity studying CXCR2 and TRPV1 channels [52], the MAPK/NF-κB [53] pathway, and the level of COX-2 expression [54], which plays important roles in cyclophosphamide-induced HC. These assays will undoubtedly decisively contribute to confirming the mechanisms underlying the IPG-induced uroprotective properties.

## 5. Conclusions

The present study provided evidence that IPG exerts anti-inflammatory and antioxidant activities which modulate the hallmarks underlying hemorrhagic cystitis, as it reduced the levels of TNF-α, IL-1β, MDA, and C-reactive protein, as well as increasing SOD levels and reducing edema and hemorrhage. These data imply that IPG represents a promising therapeutic option in the treatment of this pathological condition, as well as that it may act as a promising co-adjuvant agent in oxazaphosphorins-based chemotherapy.

## Data Availability

Not applicable.
